# Social media’s enduring effect on adolescent life satisfaction

**DOI:** 10.1073/pnas.1902058116

**Published:** 2019-05-06

**Authors:** Amy Orben, Tobias Dienlin, Andrew K. Przybylski

**Affiliations:** ^a^Department of Experimental Psychology, University of Oxford, Oxford OX2 6GG, United Kingdom;; ^b^Oxford Internet Institute, University of Oxford, Oxford OX1 3JS, United Kingdom;; ^c^Department of Media Psychology, University of Hohenheim, 70593 Stuttgart, Germany

**Keywords:** social media, adolescents, life satisfaction, longitudinal, random-intercept cross-lagged panel models

## Abstract

In this study, we used large-scale representative panel data to disentangle the between-person and within-person relations linking adolescent social media use and well-being. We found that social media use is not, in and of itself, a strong predictor of life satisfaction across the adolescent population. Instead, social media effects are nuanced, small at best, reciprocal over time, gender specific, and contingent on analytic methods.

Does the increasing amount of time adolescents devote to social media negatively affect their satisfaction with life? Set against the rapid pace of technological innovation, this simple question has grown into a pressing concern for scientists, caregivers, and policymakers. Research, however, has not kept pace (1). Focused on cross-sectional relations, scientists have few means of parsing longitudinal effects from artifacts introduced by common statistical modeling methodologies (2). Furthermore, the volume of data under analysis, paired with unchecked analytical flexibility, enables selective research reporting, biasing the literature toward statistically significant effects (3, 4). Nevertheless, trivial trends are routinely overinterpreted by those under increasing pressure to rapidly craft evidence-based policies.

Our understanding of social media effects is predominately shaped by analyses of cross-sectional associations between social media use measures and self-reported youth outcomes. Studies highlight modest negative correlations (3), but many of their conclusions are problematic. It is not tenable to assume that observations of between-person associations—comparing different people at the same time point—translate into within-person effects—tracking an individual, and what affects them, over time (2). Drawing this flawed inference risks misinforming the public or shaping policy on the basis of unsuitable evidence.

To disentangle between-person associations from within-person effects, we analyzed an eight-wave, large-scale, and nationally representative panel dataset (Understanding Society, the UK Household Longitudinal Study, 2009–2016) using random-intercept cross-lagged panel models (2). We adopted a specification curve analysis framework (3, 5)—a computational method which minimizes the risk that a specific profile of analytical decisions yields false-positive results. In place of a single model, we tested a wide range of theoretically grounded analysis options [data is available on the UK data service (6); code is available on the Open Science Framework (7)]. The University of Essex Ethics Committee has approved all data collection on Understanding Society main study and innovation panel waves, including asking consent for all data linkages except to health records not used in this study.

While 12,672 10- to 15-y-olds took part, the precise number of participants for any analysis varied by age and whether full or imputed data were used (range, *n* = 539 to 5,492; median, *n* = 1,699). Variables included (*i*) a social media use measure: “How many hours do you spend chatting or interacting with friends through a social website like [Bebo, Facebook, Myspace] on a normal school day?” (5-point scale); (*ii*) six statements reflecting different life satisfaction domains (7-point visual analog scale); and (*iii*) seven child-, caregiver-, and household-level control variables used in prior work (3). We report standardized coefficients for all 2,268 distinct analysis options considered.

We first examined between-person associations (Fig. 1, *Left*), addressing the question Do adolescents using more social media show different levels of life satisfaction compared with adolescents using less? Across all operationalizations, the median cross-sectional correlation was negative (ψ = −0.13), an effect judged as small by behavioral scientists (8). Next, we examined the within-person effects of social media use on life satisfaction (Fig. 1, *Center*) and of life satisfaction on social media use (Fig. 1, *Right*), asking the questions Does an adolescent using social media more than they do on average drive subsequent changes in life satisfaction? and To what extent is the relation reciprocal? Both median longitudinal effects were trivial in size (social media predicting life satisfaction, β = −0.05; life satisfaction predicting social media use, β = −0.02).

**Fig. 1.**
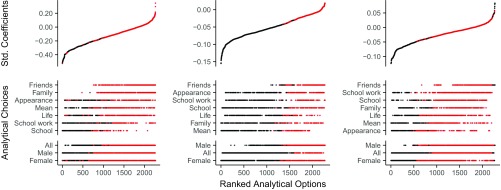
Results of a random-intercept cross-lagged panel model specification curve analysis relating social media use and life satisfaction. (*Left*) Between-person correlations. (*Center*) Within-person effects of social media use on life satisfaction. (*Right*) Within-person effects of life satisfaction on social media use. Each point on the *x* axis represents a different combination of analytical decisions (i.e., life satisfaction domain, gender, number of waves, estimator, data imputation, and control variables). The “dashboard” depicts which gender and life satisfaction domain the specific combination of analytical decisions analyzed; the resulting ψ value (*Left*) or β value (*Center* and *Right*) is shown in the plot above (red indicates *P* > 0.05, black indicates *P* < 0.05). For the unabridged figure, including the complete set of analytic decisions and underlying code, see doi.org/10.17605/OSF.IO/4XP3V.

When examining the range of possible specifications, the importance of gender was apparent: Only 16% of significant models arose from male data. Across most models (Fig. 1), the median between-person relation and within-person effects appeared more negative for females, hinting that gender was playing an underexplored role in the influence of social media. We therefore conducted a focused analysis of individual models informed by the extant social media effects literature, statistical best practices, and the data characteristics. Each model (*i*) considered female and male participants separately, (*ii*) included all relevant covariates, (*iii*) used weighted least squares mean and variance adjusted estimators to account for ordinal scales, (*iv*) implemented predictive mean matching to impute missing data (9), and (*v*) used data from participants who completed four waves, as fewer would be the absolute minimum necessary for our models (2) and examining more would decrease participant numbers, thereby reducing robustness.

Although some significant between-person correlations for both genders were in evidence (Fig. 2, *Left*), males showed only two longitudinal within-person effects: Social media predicted tenuous decreases in satisfaction with life and in mean satisfaction (*b* = −0.08 to −0.04 or β = −0.07 to −0.05; Fig. 2, *Center*). For females, however, social media was a predictor of slightly decreased life satisfaction across all domains, except satisfaction with appearance (*b* = −0.13 to −0.05 or β = −0.09 to −0.04; Fig. 2, *Center*). Furthermore, all domains of life satisfaction, except satisfaction with friends, predicted slightly reduced social media use (*b* = −0.17 to −0.05 or β = −0.11 to −0.07; Fig. 2, *Right*).

**Fig. 2.**
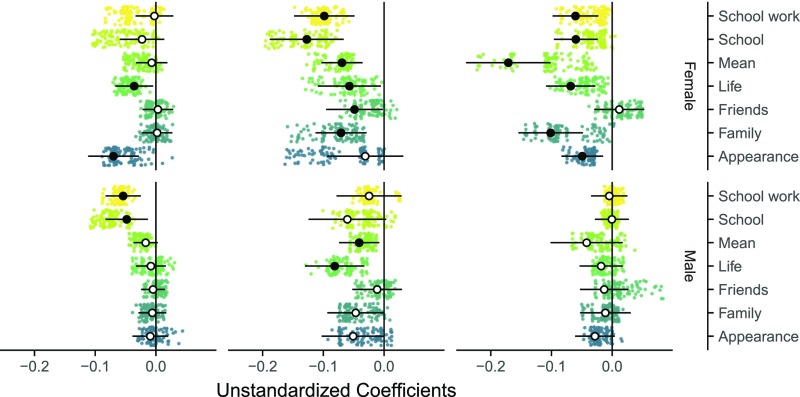
Relation between life satisfaction and social media use in female (*Upper*) and male (*Lower*) adolescents. (*Left*) Between-person correlations. (*Center*) Within-person effects of social media use on life satisfaction. (*Right*) Within-person effects of life satisfaction on social media use. Small dots represent results of each possible combination of theoretically defensible analytical decisions. Large circles represent results of the best practice models (white indicates *P* > 0.05, black indicates *P* < 0.05).

However, some caution is warranted: When comparing both genders, the effects’ confidence intervals overlap, and the lower incidence of significant effects in males alone is not evidence that the effects are therefore substantial in females (10), especially as they are very small in size. Further, the yearly interval between measurements in these data might not be optimal for understanding reciprocal social media effects over time, underlining how no single study can capture the full causal picture. We also highlight that self-report measures only partially reflect the objective time adolescents spend engaging with social media (11), yet they form the foundation of technological assessments included in the best-quality datasets informing vital research in this area today.

The relations linking social media use and life satisfaction are, therefore, more nuanced than previously assumed: They are inconsistent, possibly contingent on gender, and vary substantively depending on how the data are analyzed. Most effects are tiny—arguably trivial; where best statistical practices are followed, they are not statistically significant in more than half of models. That understood, some effects are worthy of further exploration and replication: There might be small reciprocal within-person effects in females, with increases in life satisfaction predicting slightly lower social media use, and increases in social media use predicting tenuous decreases in life satisfaction.

With the unknowns of social media effects still substantially outnumbering the knowns, it is critical that independent scientists, policymakers, and industry researchers cooperate more closely. Scientists must embrace circumspection, transparency, and robust ways of working that safeguard against bias and analytical flexibility. Doing so will provide parents and policymakers with the reliable insights they need on a topic most often characterized by unfounded media hype. Finally, and most importantly, social media companies must support independent research by sharing granular user engagement data and participating in large-scale team-based open science. Only then will we truly unravel the complex constellations of effects shaping young people in the digital age.
